# Eryptosis in Patients with Chronic Kidney Disease: A Possible Relationship with Oxidative Stress and Inflammatory Markers

**DOI:** 10.3390/jcm11237167

**Published:** 2022-12-02

**Authors:** Anna Clementi, Grazia Maria Virzì, Sabrina Milan Manani, Giovanni Giorgio Battaglia, Claudio Ronco, Monica Zanella

**Affiliations:** 1 Department of Nephrology and Dialysis, Santa Marta and Santa Venera Hospital, 95024 Acireale, Italy; 2 International Renal Research Institute (IRRIV), 36100 Vicenza, Italy; 3 Department of Nephrology, Dialysis and Transplant, St Bortolo Hospital, 36100 Vicenza, Italy

**Keywords:** eryptosis, oxidative stress, red blood cells, CKD, inflammation

## Abstract

Background. Eryptosis is the programmed death of red blood cells; it may contribute to worsening anemia in chronic kidney disease (CKD). In this clinical condition, different factors induce eryptosis, such as oxidative stress, energy depletion and uremic toxins. In our study, we investigated if the progression of CKD may influence erythrocyte death levels and its relationship with oxidative stress and inflammation. Methods. We evaluated eryptosis levels in 25 CKD patients (five for each stage), as well as markers of oxidative stress and inflammation: myeloperoxidase (MPO), copper/zinc superoxide dismutase (Cu/Zn SOD) and interleukin-6 (IL-6) were evaluated in plasma samples. Results. Higher cell death rate was reported in the highest CKD stages (*p* < 0.05). Furthermore, we divided CKD patients into two groups (eGFR< or ≥60 mL/min/1.73 m^2^). Patients with eGFR < 60 mL/min/1.73 m^2^ had higher eryptosis levels (*p* < 0.001). MPO, CU/Zn SOD and IL-6 resulted significantly differently between groups (*p* < 0.001). Significant positive correlations were reported between eryptosis and MPO (Spearman’s rho = 0.77, *p* = 0.01) and IL-6 (Spearman’s rho = 0.52, *p* = 0.05) and Cu/Zn SOD. Spearman’s rho = 0.6, *p* = 0.03). Conclusions. In patients with CKD, different factors are involved in the pathogenesis of eryptosis, in particular uremic toxins and oxidative stress and inflammatory markers. The progressive impairment of renal function may be associated with the increase in eryptosis levels, probably due to the accumulation of oxidative stress factors, inflammatory cytokines and uremic toxins.

## 1. Introduction

Even though anemia in chronic kidney disease (CKD) results mainly from the decreased production of erythropoietin, eryptosis seems to be a contributing factor. Eryptosis, which is the programmed death of red blood cells (RBCs), is characterized by cell shrinkage and membrane scrambling with phosphatidylserine (PS) exposure at the erythrocyte surface [1]. These modifications are induced by calcium influx into the RBCs activated by prostaglandin E2 release and oxidative stress [2]. The activation of calcium channels is responsible for the increase of cytosolic calcium, which in turn induces K^+^ efflux through Ca^2+^-activated K^+^ channel K_Ca_ 3.1, and the exposure of phosphatidylserine on the cell surface with subsequent degradation of RBCs by macrophages [3,4]. Indeed, phagocytic cells recognize phosphatidylserine on the cell surface, thus removing RBCs from circulation through a receptor-mediated mechanism. This process may contribute to worsening anemia in CKD [5,6].

It has been demonstrated that, in patients undergoing hemodialysis, eryptosis levels are higher compared with healthy individuals [7]. In patients with CKD, different factors may contribute to increase eryptosis, such as oxidative stress, energy depletion and uremic toxins. In particular, different studies have demonstrated that uremic toxins may trigger eryptosis, such as indoxyl sulphate [8], acrolein [9] and indole-3-acetic acid [10].

Moreover, in CKD uremic toxins and reactive oxygen species (ROS) promote inflammation and oxidative stress through the stimulation of polymorphonuclear lymphocytes, leading to the release of inflammatory cytokines. End-stage renal disease is also characterized by a suppression of the antioxidant system which exacerbates oxidative stress. All these factors are responsible for the impairment of RBC membrane structures in patients with CKD [11,12].

In this study, we investigated if the progression of CKD may influence erythrocyte death levels and its relationship with oxidative stress and inflammation.

## 2. Materials and Methods

### 2.1. Enrollment and Blood Collection

Our study was approved by the ethics committee of San Bortolo Hospital in Vicenza (n° 58/17). In this in vitro study, we evaluated eryptosis levels in 25 CKD patients (5 for each stage). Dialysis patients were excluded. We recruited blood samples from 25 CKD patients (5 for each stage) who came to our center (Nephrology Department at San Bortolo Hospital in Vicenza, Italy) for a routine check-up. CKD stages were defined according to the KDIGO (Kidney Disease Improving Global Outcomes) 2012 guidelines [13]. In particular, in stage 5 there were no dialysis patients. CKD patients with serious medical comorbidities, including active infection, autoimmune disease, and malignant, pulmonary or hepatic disease were excluded from the study. In addition, CKD patients were divided in 2 groups based on eGFR (estimated glomerular filtration rate) levels (eGFR> or <60 mL/min/1.73 m^2^). Moreover, CKD patients were divided into three groups: normal renal function, CKD with GFR ≥ 60 mL/min/1.73 m^2^ and CKD with GFR < 60 mL/min/1.73 m^2^. Serum creatinine (SCr) was measured by an enzymatic method, isotope dilution mass spectrometry traceable by an automatic analyzer (Dimension Vista; Siemens Healthcare, Tarrytown, NY, USA), and eGFR was calculated with the CKD-EPI equation [14]. Demographic and clinical characteristics and laboratory parameters were recorded for all patients.

Haemoglobin (Hb), heamatocrit (Hct), ferrum (Fe) and ferritin have been evaluated.

Blood was collected in one single 4.5 mL ethylenediaminatetra-acetic acid (EDTA tube) and processed within 1 h after venipuncture. Samples were centrifuged for 10 min at 1600× *g*. We tested all samples with hemolysis index to avoid samples with hemolysis that could confuse subsequent analysis. This blood collection was performed by a medical doctor and the handling of samples was performed under highly strict aseptic conditions, in order to prevent any contamination of samples.

### 2.2. Eryptosis Evaluation

For the CKD population, we directly measured eryptosis in freshly isolated RBCs from the different groups in parallel. PS avidly binds Annexin-V, which is thus employed to identify eryptotic cells. Thus, PS exposure at RBC surface was estimated from FITC-AnnexinV binding using flow cytometric analyses. 1 μL of isolated RBCs was washed in 400 μL Ringer solution containing 2 mM CaCl_2_ and then stained with 1 μL of AnnexinV-FITC-conjugated (Beckman Coulter, Brea, CA, USA) for 20 min in the dark. Then 400 μL of Ringer was added to each tube. Analysis was performed by Navios Flow Cytometer (Beckman Coulter, Brea, CA, USA) to identify the subpopulations of the eryptotic RBCs within 1 h. RBCs were gated and enumerated by identifying those cells that exposured PS at RBC surface. In the following, the forward scatter (FSC) of the cells was determined, and Annexin-V fluorescence intensity was measured with an excitation wavelength of 488 nm and an emission wavelength of 530 nm. A minimum 100,000 events were collected on each sample.

### 2.3. Oxidative Stress Detection

A quantitative determination of oxidative stress was performed in the plasma samples of all CKD patients.

#### 2.3.1. Myeloperoxidase (MPO) Enzyme-Linked Immunosorbent Assay (ELISA) Detection

Quantitative determination of plasma MPO concentration was performed by Human Instant ELISA kit (eBioscience, San Diego, CA, USA). Preliminary plasma dilution 1:100 was performed for each sample with specific Sample Diluent (eBioscience, San Diego, CA, USA). MPO determination was performed according to the manufacturer’s protocol and instructions. Optical density was read by using a VICTORX4 Multilabel Plate Reader (PerkinElmer Life Sciences, Waltham, MA, USA) at 450 nm. The levels of this molecule were calculatedfrom the standard curve based on the manufacturer’s protocol. Standard samples ranged from 0.16–10.0 ng/mL. Human MPO Instant ELISA Kit sensitivity is 0.03 pg/mL. All tests were performed in triplicate.

#### 2.3.2. Copper/Zinc Superoxide Dismutase (Cu/ZnSOD) ELISA Detection

Quantitative determination of Cu/ZnSOD concentration in plasma samples was performed by Human ELISA kit (eBioscience, San Diego, CA, USA). Preliminary plasma dilution 1:20 was performed for each sample with specific Sample Diluent (eBioscience, San Diego, CA, USA). Cu/ZnSOD determination was performed based on manufacturer’s protocol and instructions. Optical density was read by using a VICTORX4 Multilabel Plate Reader (PerkinElmer Life Sciences, Waltham, MA, USA) at 450 nm. The levels of these molecules were calculated from the standard curve according to the manufacturer’s protocol. Standard samples ranged from 0.08–5.0 ng/mL. Human Cu/ZnSODInstant ELISA Kit sensitivity is 0.04 ng/mL. All the tests were performed in triplicate.

### 2.4. Inflammation Evaluation: IL-6 Detection

Quantitative determination of IL-6 in plasma samples was performed by Human Instant ELISA kit (eBioscience, San Diego, CA, USA). Cytokine determination was performed according to manufacturer’s protocol and instructions. Optical density was read by using a VICTORX4 Multilabel Plate Reader (PerkinElmer Life Sciences, Waltham, MA, USA) at 450 nm. The levels of this molecule were calculated from standard curves, according to the manufacturer’s protocol. All the tests were performed in triplicate. Standard samples for IL-6 ranged from 3.1 to 200 ng/mL and the sensitivity of this test was 0.92 ng/mL.

### 2.5. Statistical Analysis

Statistical analysis was performed using the SPSS Software package and Excel. A *p*-value of <0.05 was considered statistically significant. Results are presented as percentages, or media and standard deviation (parametric variables) or medians and interquartile ranges (nonparametric variables). The Mann–Whitney U test or T test was used for comparison of the two groups when appropriate. The Kruskal–Wallis test or ANOVA test for multiple comparisons were applied to compare the groups when appropriate. Correlation coefficients were calculated with the Spearman’s rank or Pearson’s test, as appropriate.

## 3. Results

A total of 25 CKD patients were enrolled in this study. Fourteenpatients were male and 11 were females, with a mean age of 57 ± 17 years old. In the study population, end-stage renal disease (ESRD) was attributed to diabetic nephropathy (seven patients), to hypertension (eight patients), nephroangiosclerosis (three patients), polycystic kidney disease (two patients), other causes (three patients) or unknown causes (two patients).

Demographic features of all 25 patients and kidney diseases responsible for CKD are listed in Table 1.

Table 2 reports medium Egfrand biochemical parameters for each stage of CKD.

Eryptosis is characterized by cell shrinkage, cell membrane scrambling and PS exposure at RBC surface. In order to investigate cell membrane scrambling, cell shrinkage and PS exposure at RBC surface, eryptotic RBCs were identified by FS (cell volume dimension) and AnnexinV binding using flow cytometric analyses.

**Figure 1:**
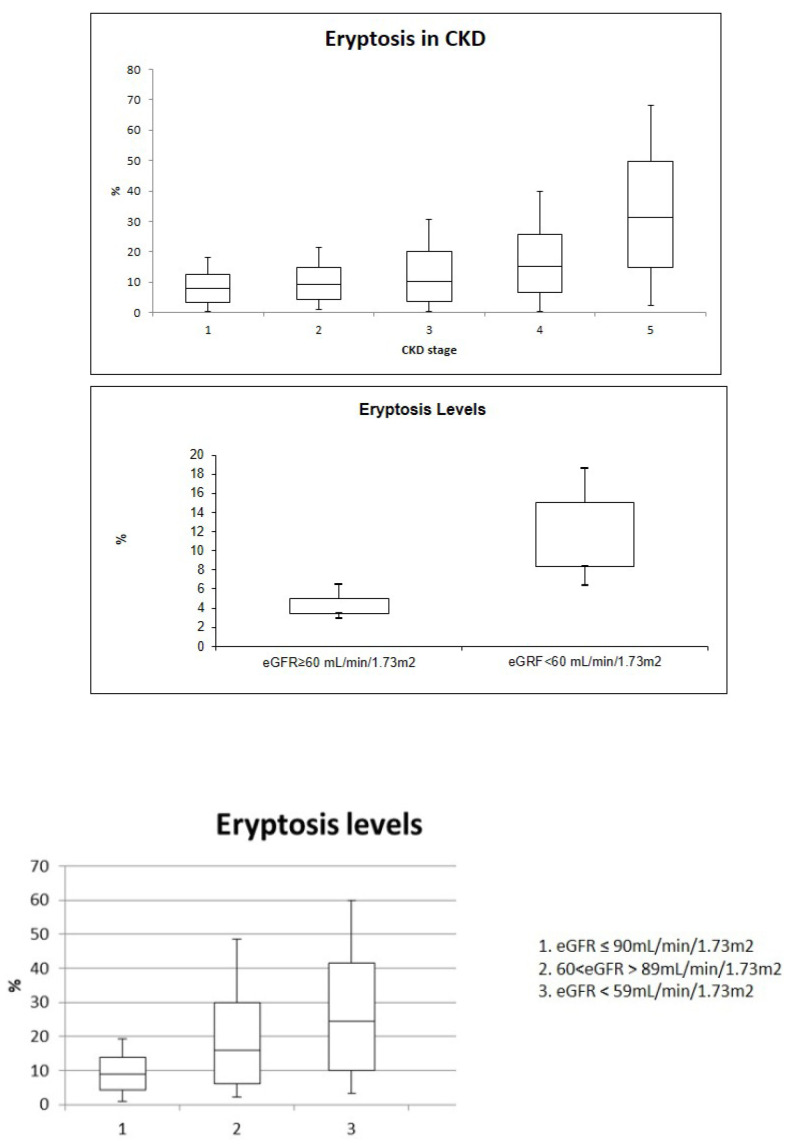
Eryptosis levels increase with worsening of Chronic Kidney Disease.

RBCs of CKD patients were dramatically deranged in their morphology and the average FS, reflecting cell volume, was significantly higher in erythrocytes from patients with CKD stage G4 and G5 (*p* < 0.001). In Figure 1 eryptosis levels are reported with a significant difference between the groups (*p* < 0.001) (Figure 1). In particular, cytofluorimetric analysis of AnnexinV highlighted significantly higher cell death rate for RBCs from patients with highest CKD stages (*p* < 0.05). Eryptosis resulted lower in RBCs from patients with CKD stage G1, while it was higher in RBCs from patients with CKD stage G5. Similar levels of eryptosis were reported between CKD G1 and CKD G2 and CKD G3. In addition, eryptosis levels resulted similarly between CKD G2 and CKD G3 and CKD G3 and CKD G4. Eryptosis levels resulted significantly higher in CKD G5 compared with all CKD stages (*p* < 0.001). Furthermore, we divided CKD patients intotwogroups (eGFR< or ≥60 mL/min/1.73 m^2^). Patients with eGFR < 60 mL/min/1.73 m^2^ were characterized by higher eryptosis levels (*p* < 0.001) (Figure 1). Moreover, we divided patients into three groups based on eGFR value. Patients with eGFR ≥ 90mL7min/1.73 m^2^ (condition similar to healthy subjects) showed eryptosis levels significantly lower than other groups (*p* < 0.001).

Furthermore, we evaluated the correlation between eryptosis and biochemical parameters in our study population. There was a significant negative correlation between eryptosis and eGFR, Hb, Hct, Fe and ferritin values (all *p* < 0.05) (Table 3).

**Figure 2:**
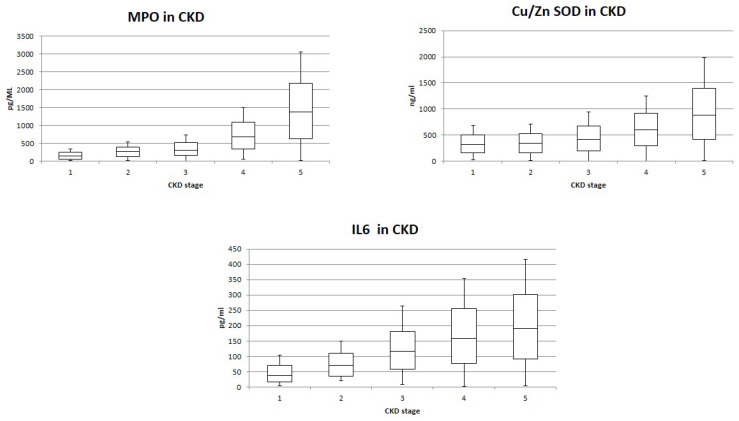
MPO, CU/Zn SOD and IL-6 levels in different CKD stages.

**Figure 3:**
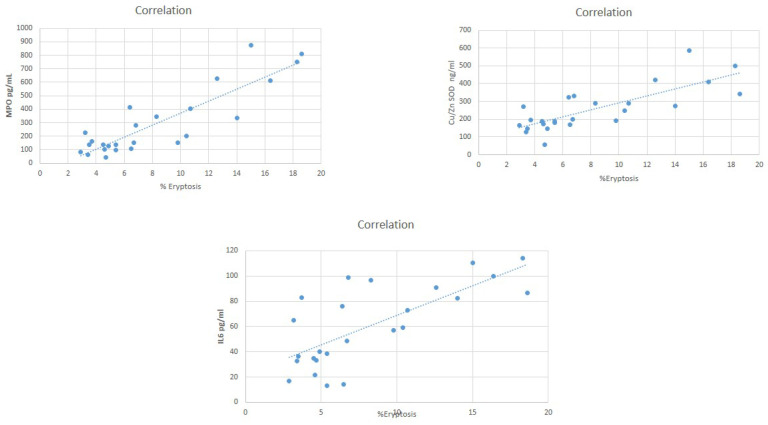
Correlation between MPO, CU/Zn SOD, IL-6 and eryptosis.

Figure 2 reports oxidative stress and IL-6 levels in our study population: MPO, CU/Zn SOD and IL-6 resulted significantly differently between groups (*p* < 0.001) (Figure 2). MPO and IL-6 differed significantly in the comparison between groups (all, *p* < 0.05). Cu/Zn SOD levels resulted similarly in CKD patients in stage G1 and G2 (*p* = 0.36) and significantly differently in other comparison between stages (all, *p* < 0.05). We observed a significant positive correlation between eryptosis and MPO (Spearman’s rho = 0.77, *p* = 0.01) and IL-6 (Spearman’s rho = 0.52, *p* = 0.05) and Cu/Zn SOD. Spearman’s rho = 0.6, *p* = 0.03) (Figure 3).

## 4. Discussion

In this study, eryptosis levels were higher in patients with advanced chronic kidney disease (stage G4 and stage G5) compared to patients with early stages of chronic renal damage (stage G1, G2 and G3). In addition, we decided to divide patients into three groups (normal renal function; CKD patients with GFR ≥ 60 mL/min/1.73 m^2^ and CKD patients with GFR < 60 mL/min/1.73 m^2^) to compare eryptosis levels in patients with mild CKD (G1) with patients with more severe CKD, since in our study a healthy population is missing. Even though patients with CKD G1 showed lower levels of eryptosis compared with patients with more severe CKD, they had higher levels of eryptosis compared to healthy individuals in a previous study (historical control group: eryptosis value: 0.8%; IQR 0.7–1.3) [15].

Indeed, different factors influence eryptosis levels in chronic kidney disease, such as oxidative stress, energy depletion, and uremic toxins [11]. All of them increase with the progressive impairment of kidney function, thus stimulating RBCs’ death levels.

In particular, uremic toxins have been associated with increased levels of eryptosis in chronic kidney disease. Indoxyl sulfate has been reported to increase cytosolic calcium concentration and to stimulate erythrocyte cell membrane scrambling, thus resulting in phosphatidylserine exposure on the surface of RBCs [8]. In the same study, indoxyl sulfate increased the levels of ceramide, which is a known contributing factor to eryptosis [8]. Similarly, acrolein seems to stimulate ceramide formation, which is responsible for the increase in cytosolic calcium concentration and eryptosis levels [9]. Moreover, in patients with chronic kidney disease, vanadate has also been demonstrated to induce eryptosis by inhibiting ATP production, thus creating an energy-deficient state [16]. This uremic toxin is also able to inhibit glycolysis within RBCs [16]. In a recent study, the cytotoxic effect of uremic toxins, such as urea and p-cresol, on healthy RBCs has been reported [17].

In our study, we reported higher levels of markers of oxidative stress and inflammation, MPO, CU/Zn SOD and IL-6, in more severe stages of chronic kidney disease (stages G4 and G5). A positive correlation between these molecules and eryptosis levels was also demonstrated.

Our data are supported by previous studies, which have shown that oxidative stress is inversely correlated with glomerular filtration rate and directly with duration of dialysis [18]. In end-stage renal disease, oxidative stress is exacerbated because of the suppressed anti-oxidant systems (decreased levels of vitamin C and glutatione) and factors increasing oxidant molecules, such as advanced age, diabetes, hypertension and dyslipidemia [19]. Specifically, hyperglycemia leads to overproduction of ROS, which increase oxidative stress, involved in the pathogenesis of diabetic nephropathy [20]. The presence of both diabetes and CKD has been shown to increase oxidative stress and eryptosis [21].

Moreover, oxidant species can also directly cause renal ischemia and glomerular damage, thus contributing to the progression of renal damage [19]. Hemodialysis itself also increases oxidative stress by the activation of the complement pathway and the stimulation of inflammatory processes [22]. Moreover, the concurrent presence of hypoxia and uremia has been recently demonstrated to augment RBC death, thus contributing to the genesis of anemia in dialysis patients [23].

Oxidative stress is responsible for the impairment of RBC membrane structures. ROS induce an injury to the lipids and proteins of RBC membranes, thus resulting in the rearrangement of the erythrocyte skeleton and the reduction of cell membrane stability and deformability [24]. Moreover, oxidative stress induces the activity of microvescicles and activates caspases, all of which aggravate eryptosis that eventually leads to renal anemia [11].

Uremic toxins and ROS promote inflammation and oxidative stress through the stimulation of polymorphonuclear lymphocytes, thus inducing the release of inflammatory cytokines, such as interleukin-1β and interleukin-8, tumor necrosis factor α [25].

The positive correlation between MPO, CU/Zn SOD and IL-6 and eryptosis levels in our study population highlights the role of oxidative stress and inflammatory cytokines in the pathogenesis of RBCs’ programmed death in patients with chronic kidney disease.

Our study has some limitations. First of all, the small number of subjects in each group and the lack of a healthy population as control group. Second, we are not able to account for other potential confounders, such age. For these reasons, our data may show an association between the progression of CKD and erythrocyte death levels and its relationship with oxidative stress and inflammation.

## 5. Conclusions

In patients with chronic kidney disease, different factors are involved in the pathogenesis of eryptosis, in particular uremic toxins and oxidative stress and inflammatory markers. The progressive impairment of renal function seems to be associated with the increase in eryptosis levels, probably due to the accumulation of oxidative stress factors, inflamamtory cytokines and uremic toxins. The identification of the factors responsible for RBCs’ programmed death may represent an important tool in the management of renal anemia in patients with chronic kidney disease.

## Figures and Tables

**Table 1:** Characteristics of the study population.

Male/Female	14 M/11 F
Age, years	57 ± 17
Diabetic nephropathy	7/25
Hypertension	8/25
Nephroangiosclerosis	3/25
Polycystic kidney disease	2/25
Other causes	3/25
Unknown causes	2/25

**Table 2:** Medium eGFR and biochemical parameters in the different CKD stages.

Chronic Kidney Disease Stages	eGFR, mL/min/1.73 m^2^	Hb, g/dL	Hct, %	Fe, µg/dL	Ferritin, ng/mL
G1	95 ± 5	12.2 ± 0.9	37.8± 0.8	75 ± 7	152 ± 10
G2	74 ±8	11.7 ± 1.4	37.2 ± 1.0	71 ± 8	133 ± 12
G3	50 ± 6	11.4 ± 1.5	35.6 ± 1.3	64 ± 12	119 ± 15
G4	24 ± 4	11.1 ± 1.4	35.1 ± 1.1	40 ± 7	122 ± 12
G5	13 ± 2	10.4 ± 1.8	33.8 ± 2.0	34 ± 6	116 ± 17

**Table 3:** Correlation between Eryptosis and eGFR, Hb, Hct, Fe and ferritin values (all, *p* < 0.05).

	Correlation Values
Eryptosis/eGFR	−0.76
Eryptosis/Hb	−0.49
Eryptosis/Hct	−0.64
Eryptosis/Fe	−0.75
Eryptosis/ferritin	−0.45

## Data Availability

All data generated or analyzed during this study are included in this article. Further inquiries can be directed to the corresponding author.

## References

[1] Lang F., Quadri S.M. (2012). Mechanisms and significance of eryptosis, the suicidal death of erythrocytes. Blood Purif..

[2] Lang F., Huber S.M., Szabo I., Gulbins E. (2007). Plasma membrane ion channels in suicidal cell death. Arch. Biochem. Biophysl..

[3] Föller M., Lang F. (2020). Ion Transport in Eryptosis, the Suicidal Death ofErythrocytes. Front. Cell Dev. Biol..

[4] Lang F., Lang E., Fller M. (2012). Physiology and pathophysiology of eryptosis. Trans. Med. Hemotherapy.

[5] Bonomini M., Sirolli V., Reale M., Arduini A. (2001). Involvement of phosphatidylserine exposure in the recognition and phagocytosis of uremic erythrocytes. Am. J. Kid. Dis..

[6] Bonan N.B., Steiner T.M., Kuntsevich V., Virzì G.M., Azevedo M., Nakao L.S., Barreto F.C., Ronco C., Thijssen A., Kotanko P. (2016). Uremic toxicity-induced eryptosis and monocyte modulation: The erythrophagocytosis as a novel pathway to renal anemia. Blood Purif..

[7] Bonomini M., Sirolli V., Settefrati N., Dottori S., Di Liberato L., Arduini A. (1999). Increased erythrocyte phosphatidylserine exposure in chronic renal failure. J. Am. Soc. Nephrol..

[8] Ahmed M.S., Abed M., Voelkl J., Lang F. (2013). Triggering of suicidal erythrocyte death by uremic toxin indoxyl sulphate. BMC Nephrol..

[9] Ahmed M.S., Langer H., Abed M., Voelkl J., Lang F. (2013). The uremic toxin acrolein promotes suicidal erythrocyte death. Kidney Blood Press. Res..

[10] Gao C., Ji S., Dong W., Qi Y., Song W., Cui D., Shi J. (2015). Indolic uremic solutes enhance procoagulant activity of red blood cells through phosphatidylserine exposure and microparticle release. Toxins.

[11] Li D., Zheng X., Zhang Y., Li X., Chen X., Yin Y., Hu J., Li J., Guo M., Wang X. (2022). What Should Be Responsible for Eryptosis in Chronic Kidney Disease?. Kidney Blood Press. Res..

[12] Gok M.G., Paydas S., Boral B., Onan E., Kaya B. (2022). Evaluation of eryptosis inpatients with chronic kidney disease. Int. Urol. Nephrol..

[13] Kidney Disease: Improving Global Outcomes (KDIGO) Work Group (2013). KDIGO 2012 clinical practice guideline for the evaluation and management of chronic kidney disease. Kidney Int. Suppl..

[14] Levey A.S., Stevens L.A., Schmid C.H., Zhang Y.L., Castro A.F., Feldman H.I., Kusek J.W., Eggers P., Van Lente F., Greene T. (2009). A new equation to estimate glomerular filtration rate. Ann. Intern. Med..

[15] Virzì G.M., Milan Manani S., Clementi A., Castegnaro S., Brocca A., Riello C., de Cal M., Giuliani A., Battaglia G.G., Crepaldi C. (2019). Eryptosis Is Altered in Peritoneal Dialysis Patients. Blood Purif..

[16] Benabe J.E., Echegoyen L.A., Pastrana B., Martinez-Maldonado M. (1987). Mechanism of inhibition of glycolysis by vanadate. J. Biol. Chem..

[17] Virzì G.M., Mattiotti M., Clementi A., Milan Manani S., Battaglia G.G., Ronco C., Zanella M. (2022). In Vitro Induction of Eryptosis by Uremic Toxins and Inflammation Mediators in Healthy Red Blood Cells. J. Clin. Med..

[18] Terawaki H., Yoshimura K., Hasegawa T., Matsuyama Y., Negawa T., Yamada K., Matsushima M., Nakayama M., Hosoya T., Era S. (2004). Oxidative stress is enhanced in correlation with renal dysfunction: Examination with the redox state of albumin. Kidney Int..

[19] Locatelli F., Canaud B., Eckardt K.U., Stenvinkel P., Wanner C., Zoccali C. (2003). Oxidative stress in end-stage renal disease: An emerging threat to patient outcome. Nephrol. Dial. Transpl..

[20] Vodosek H., Bevc S., Ekart R., Hojs R. (2020). Oxidative stress markers in chronic kidney disease with emphasis on diabetic nephropathy. Antioxidants.

[21] Calderón-Salinas J.V., Muñoz-Reyes E.G., Guerrero-Romero J.F., Rodríguez-Morán M., Bracho-Riquelme R.L., Carrera-Gracia M.A., Quintanar-Escorza M.A. (2011). Eryptosis and oxidative damage in type 2 diabetic mellitus patients with chronic kidney disease. Mol. Cell Biochem..

[22] Mekki K., Taleb W., Bouzidi N., Kaddous A., Bouchenak M. (2010). Effect of hemodialysis and peritoneal dialysis on redox status in chronic renal failure patients: A comparative study. Lipids Health Dis..

[23] Tozoni S.S., Dias G.F., Bohnen G., Grobe N., Pecoits-Filho R., Kotanko P., Moreno-Amaral A.N. (2019). Uremia and Hypoxia Independently Induce Eryptosis and Erythrocyte Redox Imbalance. Cell Physiol. Biochem..

[24] Sudnitsyna J., Skverchinskaya E., Dobrylko I., Nikitina E., Gambaryan S., Mindukshev I. (2020). Microvesicle Formation Induced by Oxidative Stress in Human Erythrocytes. Antioxidants.

[25] Tbahriti H.F., Meknassi D., Moussaoui R., Messaoudi A., Zemour L., Kaddous A., Bouchenak M., Mekki K. (2013). Inflammatory status in chronic renal failure: The role of homocysteinemia and pro-inflammatory cytokines. World J. Nephrol..

